# Roles of existing drug and drug targets for COVID-19 management

**DOI:** 10.1016/j.metop.2021.100103

**Published:** 2021-06-29

**Authors:** Akeberegn Gorems Ayele, Engidaw Fentahun Enyew, Zemene Demelash Kifle

**Affiliations:** a Department of Pharmacology and Clinical Pharmacy, School of Pharmacy, College of Health Science, Addis Ababa University, Addis Ababa, Ethiopia; b Department of Human Anatomy, School of Medicine, College of Medicine and Health Sciences, Gondar, Ethiopia; c Department of Pharmacology, School of Pharmacy, College of Medicine and Health Science, University of Gondar, Gondar, Ethiopia

**Keywords:** COVID-19, Existing drug, Therapeutic target, Management, COVID-19, coronavirus disease-2019, 3CLpro, 3-chymotrypsin-like protease, AAK1, adaptor-associated kinase 1, Nsp, non-structure protein, PLpro, papain-like protease, RdRp, RNA-dependence RNA-polymerase, CEF, Cepharanthine, ACE-2, Angiotensin-Converting Enzyme-2, SARS-COV-2, severe acute respiratory syndrome coronavirus-2, TMPRSS2, transmembrane Serine Protease 2, ORF, open reading frame, TPC2, two-pore channel 2, GAK, cyclin G-associated kinase, MERS-CoV, Middle East respiratory syndrome coronavirus

## Abstract

In December 2019, a highly transmissible, pneumonia epidemic caused by a novel coronavirus, severe acute respiratory syndrome coronavirus 2 (SARS-CoV-2), erupted in China and other countries, resulting in devastation and health crisis worldwide currently. The search and using existing drugs support to curb the current highly contagious viral infection is spirally increasing since the pandemic began. This is based on these drugs had against other related RNA-viruses such as MERS–Cov, and SARS-Cov. Moreover, researchers are scrambling to identify novel drug targets and discover novel therapeutic options to vanquish the current pandemic. Since there is no definitive treatment to control Covid-19 vaccines are remain to be a lifeline. Currently, many vaccine candidates are being developed with most of them are reported to have positive results. Therapeutic targets such as helicases, transmembrane serine protease 2, cathepsin L, cyclin G-associated kinase, adaptor-associated kinase 1, two-pore channel, viral virulence factors, 3-chymotrypsin-like protease, suppression of excessive inflammatory response, inhibition of viral membrane, nucleocapsid, envelope, and accessory proteins, and inhibition of endocytosis were identified as a potential target against COVID-19 infection. This review also summarizes plant-based medicines for the treatment of COVID-19 such as *saposhnikoviae divaricata, lonicerae japonicae flos, scutellaria baicalensis, lonicera japonicae,* and some others. Thus, this review aimed to focus on the most promising therapeutic targets being repurposed against COVID-19 and viral elements that are used in COVID-19 vaccine candidates.

## 1 Introduction

Coronaviruses (CoVs) are single-stranded, positive-sense RNA viruses from the family Coronaviridae (subfamily Coronavirinae) that infect a wide variety of hosts and cause diseases ranging from the common cold to severe/fatal illnesses [1]. The virus was initially known as “2019-nCoV” before the name was changed to “SARS-CoV-2” or “Covid-19 “by Coronavirus Study Group (CSG) and World health organization (WHO) respectively [2].

COVID-19 outbreak began in October-2019 in Wuhan and has since spread across China and the rest of the world [3]. The symptoms of the deadliest viral infections can range from asymptomatic/mild symptoms to serious illness and death. Symptoms can appear after 2 days to 2 weeks after virus exposure. Among signs and symptoms fever, difficulty in breathing, cough, and muscle or body ache [4]. Globally, as of 8 April 2021, there have been 132,730,691 confirmed cases of COVID-19 with nearly 2,880,726 deaths, and due to vaccine rollout worldwide, a total of 669,248,795 vaccine doses have been administered [5].

The pandemic has been in effect for several months. And, scholars are not sure for how many years the virus will linger with a human being. On the other hand, to combat the virus, the world, is searching for a viable treatment option to combat the viral infection [6]. Although many studies on the treatment of COVID-19 infection have been conducted, there is a dearth of reports that comprehensively articulate novel targets, vaccines, and drug repurposing for tamping down COVID-19 infection across the globe [7]. This article, therefore, highlights an update on promising repurposed therapeutic candidates, novel drug targets, and ongoing advances in designing vaccines to tackle covid-19.

## 2 Drug re-purposing for COVID-19

In contrast to the de novo drug development method, drug repurposing is a process of finding potential applications for approved or investigational products. It is a very useful technique for drug discovery because it takes less time and money to find a therapeutic agent [8]. Currently, there is no definitive treatment approach for SARS-CoV-2. As a result, re-tasking drugs for a new pandemic is crucial [8,9]. This section, therefore, attempted to summarize drugs repurposed for COVID-19.

### 2.1 Interleukin-6 inhibitors

Severe COVID-19 is characterized by significantly elevated plasma levels of the proinflammatory mediators including, IL-6, suggesting that blockage of this target might improve the clinical outcome of the patient [10]. Food and Drug Administration (FDA)-approved two classes of IL-6 inhibitors: anti-IL-6 receptor monoclonal antibodies (tocilizumab, sarilumab,) and anti-IL-6 monoclonal antibodies (siltuximab). These classes of drugs have been evaluated for the management of patients with COVID-19 who have systemic inflammation [11].

#### 2.1.1 Tocilizumab

Tocilizumab is a monoclonal antibody that binds to the receptor for interleukin (IL)-6, reducing inflammation, and is commonly used to treat rheumatoid arthritis chimeric antigen receptor T cell [12]. A study conducted by Christine A. Vu *et al* showed that since tocilizumab administration to COVID-19 begun, the majority of patients showed clinical progress and were safely released alive from the hospital [13] On the contrary, however, a randomized trial study involving hospitalized patients with severe COVID-19 pneumonia showed the use of tocilizumab did not result in considerably better clinical status or lower mortality than placebo at 28 days course of therapy [14].

Currently, the Infectious Disease Society of America guidelines recommend tocilizumab in addition to standard of care (ie, steroids) in adults with COVID-19 who have elevated markers of systemic inflammation [15]. The National Institutes of Health recommend the use of tocilizumab (single IV dose of 8 mg/kg, up to 800 mg) along with dexamethasone to hospitalized patients who are exhibiting rapid respiratory decompensation caused by COVID-19 [11].

#### 2.1.2 Sarilumab

Sarilumab, like Tocilizumab, is a human monoclonal antibody that binds to and inhibits IL-6-mediated signaling and thereby lowering inflammation. Following registration by the European Medicine Agency in April 2017, Sarilumab received approval from FDA latter one month in the same year [16]. Different studies have been conducted regarding sarilumab's effect against SARS-Cov-2. A study conducted by Elisa Gremese *et al* showed that sarilumab has a good global rate of clinical outcome to treat SARS-CoV-2 severe pneumonia in terms of decreasing mortality and producing clinical improvement. This study also found that patients treated with sarilumab had a greater intensive care unit (ICU) discharge rate compared to those who did not receive it [17]. In another study sarilumab was associated with increased survival and reduced hospitalization [18].

#### 2.1.3 Siltuximab

Siltuximab has reached a phase 3 clinical trial by FDA in hospitalized patients with COVID-19 associated acute respiratory distress syndrome [19] A prospective cohort study on patients who received siltuximab revealed that siltuximab has significantly reduced risk of mortality [18].

### 2.2 Drugs acting on viral replication

#### 2.2.1 Ribavirin

Ribavirin is a guanosine analog, which was previously approved for the treatment of the severe respiratory syncytial virus, and has been used in the treatment of Lassa fever virus infection, influenza A and B, and other viruses [20]. Ribavirin interferes with the replication of RNA and DNA virus. The anti-viral activity of ribavirin is not only limited to polymerase but can also directly inhibit inosine monophosphate dehydrogenase. Through this ribavirin prevents natural guanosine generation and this pathway is a cornerstone for the production of guanosine from guanine precursor [21].

Ribavirin has a well‐established history of usage in the emergency clinical management of SARS‐CoV outbreaks in China and North America and MERS‐CoV outbreaks in Asia and the Middle East. During these outbreaks, early administration upon presentation with pneumonia and before sepsis or organ system failure has been reported to provide the greatest benefit. This led to a base for ribavirin to be used for COVID-19 and in China a guideline was implemented. Accordingly, a 4‐g oral loading dose should be delivered, followed by 1.2‐g oral dose every 8 h. This guidance was later amended to 500 mg intravenously twice or three times a day [22]. In a retrospective analysis, although ribavirin alone did not produce a good clinical outcome, the combinations of ribavirin with lopinavir-ritonavir or interferon was found to produce a favorable result in terms of reducing mortality, accelerating clinical improvement, and reducing hospital stay [23].

#### 2.2.2 Cepharanthine

Cepharanthine (CEP) is a naturally occurring alkaloid obtained from *Stephania cepharantha Hayata* that has been shown to have anti-inflammatory, antioxidative, immunomodulatory, antiparasitic, and antiviral properties. Moreover, CEP suppresses responsible components for viral replication and inflammatory response such as lipid peroxidation, nuclear factor-kappa B activation, cytokine production, nitric oxide production, and expression of cyclooxygenase suggesting possible use in a viral disease such as COVID-19. In TNF-α-stimulated U1 monocytic cells, which is an *in vitro* study, CEP at 0.016 μg/ml and cytotoxic concentration of 2.2 μg/ml inhibited the replication of the virus in a dose-dependent manner [24]. In another *in vitro* SARS-CoV-2 inoculated VeroE6/TMPRSS2 cells, CEP was found to produce an excellent antiviral effect acting predominantly through interfering with the ability of the virus to attach with its target cell via S glycoprotein [25].

In an *in silico* docking simulation study, there is a prospect that CEP molecule can bind the SARS-CoV-2 S protein, and interfere with S-protein interaction to its receptor (ACE-2). According to the docking model, the hydrogen bond will be formed by the piperidine ring of CEP molecule with its different side chain and the aromatic rings are close to contact with the binding interface of ACE 2 [26].

### 2.3 Drugs acting on viral entry

#### 2.3.1 Darunavir

Darunavir in indicated for the treatment of patients with human immunodeficiency virus in combination with other antiretroviral medicinal products. Darunavir acts by selectively inhibiting the cleavage of HIV- encoded Gag-Pol polyproteins in the virus-infected cells, thereby preventing the formation of mature infection virus cells [27]. Investigating the effects of Darunavir against COVID-19 came since drugs in the same class (lopinavir/ritonavir) were found to be effective for MERS-CoV infection in animal experiments and case reports [28]. Unfortunately, in the single-center, randomized trial, the combinations of darunavir and cobicistat in COVID-19 patients did not produce a notable negative polymerase chain reaction compared to the control group [28]. Moreover, darunavir failed to show antiviral activity against SARS-CoV-2 at clinically relevant concentrations (EC50 > 100 mM) in an *in vitro* study [29].

#### 2.3.2 Nafamostat

Nafamostat mesylate is a serine protease inhibitor that blocks proteolytic enzymes, such as thrombin, plasmin, and trypsin, and has been used in Japan for disseminated intravascular coagulation and pancreatitis treatment [30]. In a small clinical case series study, Kent Doi *et al* found that the mortality rate in COVID-19 patients treated with combinations of nafamostat and favipiravir was low suggesting these combinations might be effective for critically ill patients due to this virus. Nafamostat/favipiravir could produce an effect through blockade of virus entry and replication, as well as inhibition of pathogenic host response [31]. In another study, nafamostat demonstrated a 15 fold higher efficacy compared to camostat mesylate in inhibiting SARS -CoV - 2 S-protein -mediated entry into the host cell [32]. On the other hand, a study conducted in Kanazawa University Hospital, Japan revealed that all COVID-19 patients developed hyperkalemia after receiving a course nafamostat therapy. This study warns monitoring serum potassium values closely after nafamostat initiation in COVID-19 patients to be compulsory [30].

### 2.4 Drugs acting on cytokine release

Increased systemic cytokine production contributes to the pathophysiology of severe COVID-19 through producing acute respiratory distress syndrome and severe inflammation, which might be treated with agents that can inhibit cytokine release and storms such as ruxolitinib and baricitinib [8,33].

#### 2.4.1 Ruxolitinib

Ruxolitinib is an oral Janus kinases (JAK) inhibitor and received FDA approval in 2011 for treatment of myelofibrosis and later in 2014 for treatment of polycythemia vera in patients unresponsive or intolerant of hydroxyurea [34]. Due to increased activation of the Janus kinase pathway, it is suggested that JAK-inhibitors such as ruxolitinib might have a useful role in treating respiratory disease due to COVID-19. Ruxolitinib is a potent and selective inhibitor of JAK 1 and 2, with modest to higher selectivity against tyrosine kinase (TYK) 2 and JAK3, respectively [35]. In a control paired case series study, patients with COVID-19 who have been received ruxolitinib had a favorable health outcome with a shorter hospital stay and no significant side effects of the drug [36].

In another clinical trial study, although it was not significant COVID-19 patients receiving ruxolitinib at a dose of 5 mg twice per day with a standard of care treatment recipients had a faster clinical improvement compared to control groups. Moreover, a significant chest computed tomography improvement and faster recovery from lymphopenia were observed in treatment groups. A favorable side effect with a reduction in inflammation was an added advantage of ruxolitinib that invigorates future trials in a larger population to assess ruxolitinib in patients with COVID-19 [37].

#### 2.4.2 Baricitinib

Baricitinibt is JAK inhibitor approved to treat mild to serious cases of rheumatoid arthritis that have not responded to one or more disease-modifying antirheumatic drugs [38].A recent pilot study on the safety and clinical impact baricitinib revealed that therapy with baricitinib had significantly shortened the recovery time, with none of the patients required ICU support, and the majority of the patients were discharged [39]. In another study, which compared 4 mg daily baricitinib with hydroxychloroquine in patients with mild-to-moderate COVID-19, baricitinib was associated with a reduction in ICU admission and mortality [40]. Similarly, barcitinib produced greater improvement of respiratory function when 2 mg or 4 mg dose is given in combination with intravenous methylprednisolone (median dose 500 mg three times daily, followed by prednisolone 30 mg per day) [41].

## 3 Convalescent plasma therapy

Convalescent plasma therapy (CPT) has a long history and it has been in use since 1901 where it was implemented for patients with diphtheria. During the 1918 pandemic, serum from convalescent patients was used to treat influenza, with some apparent success [42]. CPTs can be manufactured by collecting whole blood or apheresis plasma from a convalescent donor [43].

Results from previous MERS and SARS coronavirus outbreaks indicated that CPT is healthy and may have clinical benefits, such as faster viral clearance, when given early in the disease course. Two to three weeks after infection, the vast majority of COVID-19 patients can produce circulating antibodies to various SARS-CoV-2 proteins, which can be detected using enzyme-linked immunosorbent assay or other quantitative assays and frequently correlate with the presence of neutralizing antibodies [44]. But, a systematic review and meta-analysis on the association of CPT with COVID-19 clinical outcome found that treatment with convalescent plasma was not significantly associated with decreeing mortality or producing any improvements in clinical outcomes [44]. In addition, CPT is associated with adverse events such as fever, chills, anaphylactic reaction, and acute lung injury. Moreover, the risk of transfusion-transmitted infections, such as human immunodeficiency virus, hepatitis B virus, hepatitis C virus, and syphilis cannot be overemphasized [45].

## 4 Vaccines

There are currently varieties of vaccine candidates being developed against a virus causing COVID-19 (Table 1). Vaccines that induce large quantities of high-affinity virus-neutralizing antibodies may prevent infection and avoid unfavorable effects [46]. As of WHO report, there are currently 87 and 186 vaccines in clinical and pre-clinical development respectively [47].

**Table 1:** COVID-19 candidate vaccines undergoing clinical evaluation.

Developer	Platform	Type	Current stage	Reference
Ludwig-Maximilians - University of Munich	Non-Replicating Viral Vector	MVA-SARS-2-S	Phase 1 NCT04569383	[154]
Vaxart	Non-Replicating Viral Vector	Ad5 adjuvanted Oral Vaccine platform	Phase 1 NCT04563702	[155]
Beijing Wantai Biological Pharmacy/ Xiamen University	Replicating Viral Vector	Intranasal flu-based-RBD	Phase 1 ChiCTR2000037782	[156]
University Hospital Tuebingen	Protein Subunit	SARS-CoV-2 HLA-DR peptides	Phase 1 NCT04546841	[157]
West China Hospital, Sichuan University	Protein Subunit	RBD (baculovirus production expressed in Sf9 cells)	Phase 1 ChiCTR2000037518	[158]
FBRI SRC VB VECTOR, Rospotrebnadzor, Koltsovo	Protein Subunit	Peptide	Phase 1 NCT04527575	[159]
Instituto Finlay de Vacunas, Cuba	Protein Subunit	RBD + Adjuvant	Phase 1 IFV/COR/04	[160]
ReiThera/ LEUKOCARE/ Univercells	Non-Replicating Viral Vector	Replication defective Simian Adenovirus (GRAd) encoding S	Phase 1 NCT04528641	[157]
Institute Pasteur/ Themis/ Univ. of Pittsburg CVR	Replicating Viral Vector	Measles-vector based	Phase 1 NCT04497298	[161]
Medicago Inc./ Université Laval	VLP	Plant-derived VLP	Phase 1 NCT04450004	[162]
People's Liberation Army (PLA)/Walvax Biotech	RNA	mRNA	Phase 1 ChiCTR2000034112	[163]
Medigen Vaccine Biologics Corporation/ NIAID/ Dynavax	Protein Subunit	S-2P protein + CpG 1018	Phase 1 NCT04487210	[164]
Curevac	RNA	mRNA	Phase 1 NCT04449276	[161]
Imperial College London	RNA	LNP-nCoVsaRNA	Phase 1 ISRCTN17072692	[157]
University of Queensland/ CSL/ Seqirus	Protein Subunit	Molecular clamp stabilized Spike protein with MF59 adjuvant	Phase 1 ACTRN12620000674932p	[164]
Vaxine Pty Ltd/ Medytox	Protein Subunit	Recombinant spike protein with Advax™ adjuvant	Phase 1 NCT04453852	[159]
Arcturus/Duke-NUS	RNA	mRNA	Phase 1/2 NCT04480957	[155]
Clover Biopharmaceuticals Inc.	Protein Subunit	Native like Trimeric subunit Spike Protein vaccine (SCB-2019)	Phase 1 NCT04437875	[165]
Gamaleya Research Institute	Non-Replicating Viral Vector	Adenovirus-based (Gam-COVID-Vac; Гам-КОВИД-Вак)	Phase 1 NCT04436471; NCT04437875	[166]
Osaka University/ AnGes/ Takara Bio	DNA	DNA plasmid vaccine + Adjuvant	Phase 1 JapicCTI-205328	[167]
Cadila Healthcare Limited	DNA	DNA plasmid vaccine	Phase 1/2 CTRI/2020/07/026352	[161]
Genexine Consortium	DNA	DNA Vaccine (GX-19)	Phase 1 NCT04445389	[161]
SpyBiotech/Serum Institute of India	VLP	RBD-HBsAg VLPs	Phase 1/2 ACTRN12620000817943	[168]
Research Institute for Biological Safety Problems, Rep of Kazakhstan	Inactivated	Inactivated	Phase 1/2 NCT04530357	[158]
Sanofi Pasteur/GSK	Protein Subunit	S protein (baculovirus production	Phase 1/2 NCT04537208	[169]
Institute of Medical Biology, Chinese Academy of Medical Sciences	Inactivated	Inactivated	Phase 1/2 NCT04470609 Phase 1 NCT04412538	[161]
Janssen Pharmaceutical Companies	Non-Replicating Viral Vector	Ad26.COV2. S	Phase 3 NCT04505722 Phase 1/2 NCT04436276	[170]
Novavax	Protein Subunit	Recombinant SARS CoV-2 glycoprotein nanoparticle vaccine adjuvanted with Matrix M (NVX-CoV2373)	Phase 3 2020-004123-16 Phase2b NCT04533399 Phase 1/2 NCT04368988	[169]
Inovio Pharmaceuticals / International Vaccine Institute	DNA	DNA plasmid vaccine with electroporation (INO-4800)	Phase 1/2 NCT04336410; NCT04447781	[171]
Anhui Zhifei Longcom Biopharmaceutical	Protein Subunit	Adjuvanted recombinant protein (RBD-Dimer)	Phase 2 NCT04466085 Phase 1 NCT04445194	[169]
Gamaleya Research Institute	Non-Replicating Viral Vector	Adenovirus-based (Gam-COVID-Vac; Гам-КОВИД-Вак)	Phase 3 NCT04530396 Phase 1 NCT04436471; NCT04437875	[172]
CanSino Biological Inc.	Non-Replicating Viral Vector	Adenovirus Type 5 Vector (Ad5-nCoV)	Phase 3 NCT04526990; NCT04540419 Phase 2 NCT04341389; Phase 1 NCT04313127	[171]
Beijing Institute of Biological Products/ Sinopharm	Inactivated	Inactivated (BBIBP-CorV)	Phase 3 ChiCTR2000034780 Phase 1/2 ChiCTR2000032459	[169]
Wuhan Institute of Biological Products/ Sinopharm	Inactivated	Inactivated	Phase 3 ChiCTR2000034780 Phase 1/2 ChiCTR2000031809	[161]
Sinovac	Inactivated	Inactivated + alum (CoronaVac; formerly PiCoVacc)	Phase 3 NCT04456595 Phase 1/2 NCT04383574; NCT04352608	[169]
BioNTech/ Fosun Pharma/ Pfizer	RNA	3 LNP-mRNAs (BNT162*)*	Approved	[144]
Moderna/ NIAID	RNA	LNP-encapsulated mRNA (mRNA-1273)	Approved	[173]
Johnson & Johnson's Janssen/ Janssen Pharmaceuticals Companies of Johnson & Johnson	Viral vector vaccine	Viral Vector	Approved	[174]
University of Oxford/ AstraZeneca	Non-Replicating Viral Vector	Weakened adenovirus (ChAdOx1-S; AZD1222)	Approved	[175]

The vaccines have been produced using different technical platforms, both old and new techniques, including mRNA vaccines [46]. For the production of new COVID-19 vaccines, a variety of approaches are used; the majority of these focus on the surface-exposed spike (S) glycoprotein or S protein as the primary inducer of neutralizing antibody. Besides S protein, other structural proteins such as N-protein have also been tested as vaccine targets [48].

### 4.1 S-protein based vaccine

The S protein is crucial for virus-cell receptor binding and virus-cell membrane fusion, implying that it may be a good target for COVID-19 vaccine design [49]. It is composed of a membrane-distal S1 and S2 subunit and exists in the virus envelope as a homotrimer. The S1 subunit defines receptor recognition via its receptor-binding domain (RBD), whereas the S2 subunit is responsible for membrane fusion, which is required for virus entry. Viral-neutralizing antibodies (nAbs) can target the S-protein to deter virus infection at multiple stages during the virus entry process. The RBD is the major target for nAbs that interfere with viral receptor binding [50].

The three most advanced vaccines (Pfizer/BioNTech, Oxford/AstraZeneca, and Moderna) are S-protein-based vaccines. Pfizer/BioNTech (BNT162b2) and Moderna (mRNA-1273) vaccines work by encoding the full length of SARSCoV-2 S- protein to elicit nAbs. Pfizer/BioNTech has also developed a vaccine that can only encode the RBD of the SARS-CoV-2 S-protein. On the other hand, Oxford/AstraZeneca's vaccines such as AZD1222 uses a non-replicating chimpanzee adenovirus vector [51].

### 4.2 N-protein based vaccine

The N protein is the most abundant in coronavirus and has multiple functions including signal transduction virus budding, formation of nucleocapsids, RNA replication, and mRNA transcription [52]. The concept of using N-protein against viral infection dates back to 1985 when Iwarson and colleagues used the hepatitis B core antigen to defend chimps from hepatitis B challenge [53]. Unlike S-protein, N-protein cannot make a substantial contribution to neutralizing antibody response and could not provide significant protection against SARS-CoV-2. On the other hand, due to its high immunogenicity character, it is used as a marker in diagnostic assays [54].

### 4.3 BCG-vaccine

Despite the rapid development of SARS-CoV-2 vaccines, it took several months to produce enough doses and the resources needed to vaccinate a large portion of the global population. In the meantime, in some countries, access to the Bacille Calmette-Guerin (BCG) vaccine may help to mitigate the pandemic's effects [55]. The BCG tuberculosis vaccine is a live attenuated strain derived from a Mycobacterium bovis isolate. BCG is one of the most commonly used vaccines in the world, with over 4 billion BCG-vaccinated people worldwide and an additional 100 million BCG-vaccinated newborn children per year [56].

Apart from preventing the propagated types of TB, some strains of BCG vaccine are known to induce immunity against infection caused by non-mycobacterial pathogens and non-related causative agents. This feature paves a way for a scientist to consider it in SARS-CoV-2 [56]. BCG vaccination may modulate antiinflammatory cytokine and chemokine responses, resulting in fewer hospitalizations and lesser COVID-19 events. According to studies, countries that use BCG in their national vaccination programs (BCG countries) have fewer reported COVID-19 cases per million people than countries that do not use BCG [57]. In addition to the effect of BCG vaccinated to be associated a decrease in the incidence of sickness during the COVID-19 pandemic, co-administration of BCG and SARS-CoV-2 vaccines may have synergistic protective effects, including increased efficacy and/or duration of the memory response [58].

## 5 Novel drug targets for COVID-19 therapeutics

Antiviral medications that target the SARS-CoV-2 can be grouped into two main groups, the 1^st^ class target inhibiting viral assembly or virus-host interactions [59]. The 2^nd^ class include medications that interfere with signaling pathways involved in viral replication or modulate broad-spectrum host innate immune responses. These medications could engage proteases or host receptors employed for viral entry and/or can influence the endocytosis pathway [[60], [61], [62]]. Principally, three general methods can be used for screening of therapeutic agents with antiviral activity against COVID-19: Inhibition of SARS-CoV-2 replication mediated by siRNA, High-throughput screening of compounds, and Repurposing of antiviral compounds [63].

Based on the life cycle of SARS-CoV virus, therapeutic agents against COVID-19 can be classified into five groups: targeting the viral structural proteins like the membrane, envelope, and Nucleocapsid protein thereby blocking virus repackaging [[64], [65], [66]]; target the viral endocytosis [[67], [68], [69], [70]]; neutralize the virus particle [[71], [72], [73], [74]]; restoration of host's innate immunity by the agents capable of producing virulent factors [[75], [76], [77], [78]]; and inhibition of virus binding to the host receptor by either chemical compounds or monoclonal antibodies [[79], [80], [81]].

### 5.1 Inhibition of endocytosis

Post fusion of the spike protein with Angiotensin-Converting Enzyme-2 receptor, it is notorious that the virus is ingested in the cells in a receptor and pH-dependent endocytosis [68]. Thus, targeting endocytosis can be a possible strategy for the prevention and treatment of COVID-19. AP-2- associated protein kinase 1 is responsible for the regulation of clathrin-mediated endocytosis [82]. According to library screening, Baricitinib which is Janus kinase inhibitor was synthesized as a potential drug candidate against COVID-19. Likewise, Oubain which is an inhibitor of clathrin-mediated endocytosis is being tried for the management of COVID-19 infection [39,67,83]. Numerous clinical trials are ongoing to evaluate the effect of chloroquine on SARS-CoV-2 [84]. In addition, *in vitro* study revealed that a derivative of Chloroquine, hydroxychloroquine is more potent in hindering COVID-19 infection than Chloroquine [85]. The possible mechanism of action for hydroxychloroquine may be by damaging the late stages of viral replication or endosome-mediated viral entry [86].

### 5.2 Inhibition of viral membrane, nucleocapsid, envelope, and accessory proteins

SARS-CoV-2 membrane, nucleocapsid, and envelope are critical for virus propagation and survival, and thus such structural proteins can be a potential target for COVID-19 management. Medications targeting these proteins will have negligible side effects since these viral proteins are structurally dissimilar from the host proteins. In addition to defending the viral genome, these structural proteins also have a role in suppressing the host immune system, thus providing the virus an advantage over the host [[87], [88], [89]]. The nucleocapsid protein acts to inhibits RNA interference-mediated by siRNA and RNA silencing. Several siRNA-based therapeutics agents are capable of inhibiting viral membrane, nucleocapsid, envelope protein translation, and viral replication through *in vitro* model [90,91]. Though, siRNA-based therapeutic agents are not accessible for human use because of the unattainability of reliable delivery systems and inherent stability issues [92]. The envelope protein also aids as an ion channel and this effect can be repressed by a drug called hexamethylene amiloride [93]. Additional chemical blocker PJ34 targets the exceptional ribonucleotide-binding pocket at the N-terminal domain of nucleocapsid protein [94].

It is vital to note that most of these therapeutic agents were intended for the management of COVID-19; because of the alterations in the COVID-19, such therapeutic agents may not be active as expected in the prevention and treatment of COVID-19. Inhibitors such as LJ003 and LJ001are broad-spectrum antiviral agents that impair the viral membrane by making singlet oxygen molecules and impede viral entry in the host. Regrettably, LJ001 is photo-dependent and physiologically unstable [95]. However, LJ001 describes a new group of antiviral agents, and additional investigation into this group of agents will yield hopeful findings [63].

### 5.3 Helicases

Helicases are motor proteins that are important to rearrange and separate and duplexes of nucleic acid in reactions driven by hydrolysis of ATP [96]. Nsp13, a superfamily 1 helicase, that is a multi-functional protein with a helicase domain and N-terminal metal-binding domain. The C-terminal makes a helicase domain whereas the N-terminal makes a Zn binding domain, and contributes to unraveling double-stranded RNA and DNA of the virus along the 5′–3′ direction in a nucleoside triphosphate-dependent manner. A previous study revealed that Nsp13 dependent unraveling is an indispensable progression for the transcription, translation, and replication of COVID-19 infection [97]. Thus, helicases could be a potential therapeutic agent for COVID-19 treatment. Several studies revealed various effective helicase inhibitors encoded by COVID-19 infection. Currently, helicases inhibitors such as ADKs, SSYA10-001, and bananins are on investigation for the management of COVID-19 [98].

### 5.4 3-Chymotrypsin-like protease (3CLpro)

3CLpro (Nsp5) facilitates Nsps maturation, which is important for the life cycle of the virus. Initially, 3CLpro is cleaved from poly-proteins to yield mature enzymes, and further cleaves Nsps at 11 sites to release Nsp4– Nsp16 [99]. Exhaustive study of the catalytic mechanism and structure of 3CLpro makes 3CLpro a potential target for the management of COVID-19 infection. Several small-molecule inhibitors and peptide inhibitors are targeting 3CLpro of COVID 19 [100]. Conivaptan, demeclocycline, ledipasvir, oxytetracycline, lymecycline, nicardipine, doxycycline, telmisartan, montelukast, and velpatasvir displayed significant binding affinity to 3CLpro [100]. Previous findings also showed that aprepitant, epirubicin, bepotastine, valrubicin, colistin, epoprostenol, icatibant, vapreotide, perphenazine, and caspofungin are capable of binding to the lopinavir/ritonavir-binding site on COVID-19 [101].

### 5.5 The viral virulence factors

A previous study revealed that COVID-19 has three virulence factors such as Nsp1, ORF7a, and Nsp3c that support virus immune escape and affect the host's innate immunity [102]. Nsp1 relates with the host 40S ribosomal subunit that constrains the production of type-I interferon and activates mainly host mRNA degradation [102]. ORF7a directly binds to bone marrow matrix antigen 2 and hinders its action by hindering the glycosylation of BST-2. BST-2 is crucial to block the release of newly assembled coronaviruses from host cells [103]. In contrast, Nsp3c are capable of binding with the host's adenosine diphosphate to assist in resisting of host innate immunity by COVID-19 [104]. These findings showed that Nsp1, ORF7a, Nsp3c could be possible therapeutic targets for treatment and prevention of COVID-19 [105]. Studies revealed that various natural products and clinical drugs with anti-inflammatory and anti-bacterial activities, such as cefpiramide, lymecycline, streptomycin, tetracycline, and piperacillin displayed relatively high binding affinity to Nsp1, ORF7a and Nsp3c target proteins [106].

### 5.6 Two-pore channel (TPC2)

In the endolysosomal system, the two-pore channels (TPC1−3) control the flow of calcium and sodium ions across cellular membranes [107]. These channels are voltage-gated, and after their binding with such a phosphoinositide, TPC2 is one of the main effectors of PI(3,5)P2 opening [108]. These have a role in the regulation of Ebola entry in the host cell and endolysosomal trafficking [109]. The structure TPC2 was testified by cryoEM, and gives valuable structural information about the open, apo, and closed forms of TPC2 (PDB codes: 6NQ0, 6NQ1, and 6NQ2, respectively) [110]. Recently, Selective estrogen receptor modulators and dopamine receptor antagonists were recognized as blockers of TPC2 through virtual screening. Particularly, pimozide and fluphenazine which are dopamine blockers together with selective estrogen receptor modulators such as raloxifene, tamoxifen, and clomiphene prevent EBOV infection in investigational models [111].

### 5.7 Cyclin G-Associated kinase (GAK) and adaptor-associated kinase 1 (AAK1)

The key entry pathway for COVID-19 infection is receptor-mediated endocytosis. GAK and AAK1 are host serine−threonine protein kinases that regulate intracellular viral trafficking throughout the entry, release, and assembly of various unrelated RNA viruses like rabies, dengue, hepatitis C, and Ebola virus [112,113]. AAK1 plays a significant role in receptor-mediated endocytosis through phosphorylation of adaptor protein 2, which excites the binding to cargo proteins. GAK facilitates the binding of clathrin to the trans-Golgi network and the plasma membrane [114]. GAK and AAK1 inhibitor such as baricitinib have been wished for as a potential therapeutic agent for COVID-19 treatment by decreasing the viral entry, though no investigational work has been conducted to verify its mechanism of action [9]. Furthermore, this therapeutic agents also decrease inflammation in patients with COVID-19 infection since it targets Janus kinase [115]. Nevertheless, studies reported that baricitinib exhibited a significant adverse effect, which may be not well tolerated by patients with COVID-19 [70]. In a recent study, both GAK and AAK1 share cysteine residues (C190 and C193, respectively) at equivalent positions that may be targeted by covalent inhibition, providing a potential target (selective covalent inhibitors) for COVID-19 infection [116]. Currently, optimizing valuable hits (3,5-disubstituted pyrrolo [2,3-b] pyridines) or use of exciting medication as potent GAK and AAK1 inhibitors could be a promising approach to manage COVID-19 INFECTION [117].

### 5.8 Cathepsin L

It is known that activation of SARS-CoV-2 spike protein through cleavage of proteases is the main step in viral infection. Several lysosomal cathepsins were pertinent in human coronavirus entry through endocytosis [118]. In the previous study, it has been revealed that only cathepsin L, and not calpain or cathepsin B, is participated in COVID-19 endocytosis entry [119]. In vitro study showed that use cathepsin L selective inhibitor (SID26681509) significantly reduced the entry of COVID-19 infection by 76% in HEK 293/hACE2 cells. This showed the potential role of cathepsin L for priming of COVID-19 S protein in the lysosome [120].

In the previous study, SSAA09E1 which is cathepsin L inhibitor was exhibited as a novel therapeutic agent for COVID-19 infection [121]. Since Cathepsin L inhibitors can reduce the progression of pulmonary fibrosis it could be a potential therapeutic option for the management of COVID-19 infection [122]. Moreover, targeting both transmembrane Serine Protease and cathepsin L is important to attain a synergistic effect against COVID-19 [123]. The big challenge in the discovery of cathepsin inhibitors is to attain selectivity. Thus, numerous computational methods have been established to resolve this problem via information from the identified 3D structures [124].

### 5.9 Transmembrane serine protease 2 (TMPRSS2)

Following receptor interaction, or in addition to it, different host proteases can activate the virus−host cell membrane fusion for subsequent genome delivery. The host cell surface (TMPRSS2 activates S protein present in the highly pathogenic human coronaviruses MERS-CoV and SARS-CoV [125]. Human TMPRSS2 is expressed in the epithelia of the gastrointestinal, urogenital, and respiratory tracts [126]. Cleavage of S protein by TMPRSS2 is preferred for coronavirus infection over other proteases, such as the endosomal cathepsins [127]. Recent research has confirmed that SARS-CoV-2 entry is facilitated by TMPRSS2 and the viral infection is decreased by the use of the protease inhibitor camostat [120]. Moreover, as a viral infection is enhanced by TMPRSS2, the Vero E6 cell line overexpressing TMPRSS2 has been described as a useful pharmacological tool for SARS CoV-2 research [128]. Last, but not least, TMPRSS2 is expressed in different cell types of lung tissue, increasing their vulnerability for SARS-CoV-2 infection [129].

TMPRSS2 has emerged as a useful drug target for antiviral drug discovery [130], and the lack of influenza and coronavirus infection has been confirmed in TMPRSS2 knock-out mice [125]. COVID-19 infection may find a potential therapy among different existing drugs with inhibitory activity against TMPRSS2. At the moment, the only camostat has shown *in vitro* activity against SARS-CoV-2, but other clinical drugs such as nafamostat and 4-(2-aminoethyl) benzenesulfonyl fluoride, all of them protease inhibitors [131], may offer some therapeutic options for the pandemic. Repurposing of the mucolytic agent called bromhexine, a TMPRSS2 inhibitor, has been also proposed for COVID-19 therapy [132].

Furthermore, transcriptional inhibition of TMPRSS2 has been proposed as a new therapeutic option. Using computational and experimental methods, estrogen and androgen-related compounds such as genistein, estradiol, and enzatulamide have been shown to reduce TMPRSS2 expression in different cell lines [133]. As TMPRSS2 expression in the human lungs seems to be modulated by estrogens and androgens, data suggest that the activation of estrogen pathways or inhibition of androgen pathways may be a new target for therapeutic clinical intervention for symptom amelioration in COVID-19 patients [134]. Currently, the crystal structure of TMPRSS2 is not available, and target-based drug discovery and design should be done using different homology models based on other well-known serine proteases structures [117].

### 5.10 Suppression of excessive inflammatory response

Cytokine response is critical for the host immune response and it has been testified that some patients with COVID-19 infection reveal a hyperinflammatory response, probably because of deregulated cytokine response. It was reported that patients with COVID-19 in the intensive care unit had an increased level of cytokines in their plasma as compared with non-intensive care unit COVID-19 patients, signifying that cytokine dysregulation has a key role in the disease progression of patients with COVID-19 infection [135,136]. Furthermore, COVID-19 infected patients admitted in intensive care unit exhibit amplified levels of IL6^+^CD4^+^T cells and GM-CSF as compared to intensive care unit naïve patient [137].

The abovementioned evidence indicates the likelihood that inhibition of extreme inflammatory response could decrease the severity of COVID-19 infection. Drugs from corticosteroids are known to have a significant systemic antiinflammation activity [138]. Though, the use of corticosteroids for COVID-19 patients is still controversial and needs exhaustive investigation. It has been confirmed that after the onset of COVID-19 infection, CD4^+^T Cells are triggered to produce inflammatory cytokines and GM-CSF [138,139]. This finding suggests that hindering the IL-6 receptor could decrease immune stress caused by COVID-19 infection. Consistent with this finding, a clinical trial is presently ongoing on Tocilizumab (IL-6 receptor-specific antibody) [140].

## 3 Plant-based medicines for the prevention and treatment of COVID-19 infection

Since the immune status plays a key role in patients with COVID-19 infection, an plant-based medicine, which possesses an immunomodulatory activity, may have a potential therapeutic agent for the prevention and treatment of COVID-19 infection [141,142]. Herbal medicines like Curcumin are taken orally to boost immunity and for their antiviral activities [143,144]. Previous studies also revealed that complementary and alternative medicines such as herbs, nutritional supplements, and multivitamins also have a key role in protecting from COVID-19 infection and boosting immunity [145]. Complementary and alternative medicines especially plant-based medicines may possess potential antiviral and preventive activities agents COVID-19 infection [146]. In the previous study it was indicated that there are four possible approaches for the application of plant-based medicines and dietary therapy against COVID-19 infection. These approaches include the use of essential oil (air-disinfectant) to halt aerosol transmission; the use of herbs and foods as supplements or diet to strengthen immunity and avert infection; use of a sanitizing agent to make available a disinfected environment; and use of antiviral agent by coating on masks [147].

Several herbal products have revealed antiviral effects against COVID-19 infection [148]. These herbal products include *cyrtomium fortune, ginger, glycyrrhiza uralensis, echinacea, exocarpium citri grandis, coriander seeds, pepper, cinchona, curcumin, caesalpinia spinosa extract, astragalus membranaceus, curcuma longa, tremetes robiniophila, curcuma xanthorrhiza, cumin, radix platycodonis, agastache rugosa, saposhnikoviae divaricata, lonicerae japonicae flos, scutellaria baicalensis, lonicera japonicae, tinospora cordifolia, rhizoma atractylodis macrocephalae, fructus forsythia, acacia senegal, nigella sativa, euterpe oleracea, glycyrrhizae glabra, withania somnifera, and atractylodis ahizoma* [[149], [150], [151], [152], [153]].

## 4 Limitations of the review

Even though this review has its strengths, such as the inclusion of many recently published research works and critically appraising the selected studies, it is not without limitations. The limitations of the current review include the complete reliance on previously published researches and high heterogeneity among the studies summarized, this may be due to a lack of standardized criteria. This review also has limitations, since there is a delay in indexing, some research articles published as of 15 April 2021 may not be considered. Likewise, as our retrieval time was only until this date, articles posted or published later this date, haven't been incorporated in the review. As some disease control plans, guidance/guidelines, and preprints are regularly updated, the date of the publication we take out may not be the time of their 1^st^ publication time. As well, only articles published in English were included in this review, which may acquaint with publication bias. Finally, we were incapable of accessing the full texts of eight articles despite contacting the authors. Though, compared with the total number of research articles encompassed in the review, we get ahead that the exclusion of these eight research articles is unlikely to have a major influence.

## 5 Conclusion

Extraordinary technology exchange and partnership in the area of antiviral therapeutic agent clinical trials and discovery will accelerate patient access to more reliable drugs with better therapeutic potential. New vaccines have been discovered and approved for COVID-19 infection in many countries. The search and using existing drugs help to curb the current highly contagious viral infection is spirally increasing since the pandemic began. This is based on these drugs had against other related RNA-viruses such as MERS–Cov, and SARS-Cov. Therapeutic targets such as helicases, transmembrane serine protease 2, cathepsin L, cyclin G-associated kinase, adaptor-associated kinase 1, two-pore channel, viral virulence factors, 3-chymotrypsin-like protease, suppression of excessive inflammatory response, inhibition of viral membrane, nucleocapsid, envelope, and accessory proteins, and inhibition of endocytosis were identified as a potential target against COVID-19 infection. Traditional herbal medicines are comprised of numerous ingredients that are most commonly taken orally. Currently, several herbal products have revealed antiviral effects against SARS-CoV-2.

## Funding

There is no funding to report.

## Ethical approval

Not applicable.

## Declaration of competing interest

The authors declare that they have no competing interests.
